# High-resolution magnetic resonance imaging for predicting successful recanalization in patients with chronic internal carotid artery occlusion

**DOI:** 10.3389/fneur.2022.1003800

**Published:** 2022-09-01

**Authors:** Xuan Zhang, Chun Zhou, Yue-zhou Cao, Chun-qiu Su, Hai-bin Shi, Shan-shan Lu, Sheng Liu

**Affiliations:** ^1^Department of Radiology, The First Affiliated Hospital of Nanjing Medical University, Nanjing, Jiangsu Province, China; ^2^Department of Interventional Radiology, The First Affiliated Hospital of Nanjing Medical University, Nanjing, Jiangsu Province, China

**Keywords:** internal carotid artery, occlusion, recanalization, magnetic resonance imaging, predictors

## Abstract

**Objective:**

The main aim of the study was to investigate the predictive factors of high-resolution magnetic resonance imaging (HR-MRI) for successful recanalization in patients with chronic internal carotid artery occlusion (CICAO).

**Methods:**

We included 41 consecutive patients who had CICAO and underwent recanalization attempts. The demographics, clinical data, and HR-MRI features in relation to the technique success were collected and analyzed using univariate and multivariate analyses. A score-based prediction model was constructed using a regression coefficient-based scoring method.

**Results:**

Technical success was achieved in 26 (63.4%) patients, with a complication rate of 12.2% (5/41). Based on multivariate analysis, occlusions involving ophthalmic artery segment (C6) or above (OR: 0.036; 95% confidence interval [CI]: 0.004–0.336) and nontapered stump (OR: 0.064; 95% CI: 0.007–0.591) were identified as independent negative predictors of successful recanalization in patients with CICAO. Point scores were assigned according to the model coefficients, and the patients who scored 0, 1, or 2 points had success rates of 93.33% (14/15), 66.67% (12/18), or 0% (0/8), respectively.

**Conclusion:**

HR-MRI characteristics may be valuable in identifying candidates for endovascular recanalization in patients with CICAO. Occlusions involving the C6 segment or higher, as well as nontapered stumps, were independent negative predictors of technical success.

## Introduction

Chronic internal carotid artery occlusion (CICAO) is usually defined as an occlusion lasting more than 4 weeks. Despite receiving the best available medical therapy, the overall risk of stroke events caused by compromised ipsilateral cerebral perfusion in patients with CICAO is about 6–20% (1, 2). The optimal treatment of CICAO is still unclear. Endovascular recanalization has been reported as an effective treatment for CICAO (3, 4). Successful recanalization reconstructs normal intracranial perfusion, alleviates clinical symptoms, and restores neurocognitive function (5, 6). However, the recanalization of CICAO still involves technical difficulties and potential complications. Several studies based on DSA have shown that occlusion length, stump morphology, and distal internal carotid artery (ICA) reconstituted by the ophthalmic artery or communicating artery are associated with the success rate of recanalization. Preoperative knowledge of CICAO characteristics may aid clinicians in the identification of eligible patients, better strategy selection, and early risk stratification.

Conventional luminal imaging modalities, such as time-of-flight magnetic resonance angiography (TOF-MRA) and computed tomography angiography (CTA), are commonly used in routine clinical practice to identify and assess CICAO. However, these imaging modalities focus on lumen information and reveal minimal information about wall characteristics. Meanwhile, previous studies have suggested that TOF-MRA and single-phase CTA may overestimate ICA severe stenosis as occlusion (7–10).

High-resolution magnetic resonance imaging (HR-MRI) has been proven to have excellent agreement with DSA in diagnosing CICAO and identifying occluded sites and proximal stump conditions (11). Moreover, HR-MRI provided additional information regarding the vessel wall and intraluminal status, such as the occluded location, length, etiologies of occlusion, wall collapse, and the presence of intraluminal thrombus. Chai et al. reported that HR-MRI might identify suitable candidates for endovascular recanalization (12). Yan et al. found that the diameters of the occluded ICA could predict the success rate of recanalization (13). However, few studies focus on whether the wall and lumen characteristics provided by HR-MRI are valuable for predicting successful recanalization before the procedure. In this study, we conducted a retrospective analysis of patients with CICAO who underwent HR-MRI before recanalization attempts to identify imaging predictors for technical success.

## Materials and methods

### Patients

This retrospective study was reviewed and approved by our Institutional Review Board, and the informed consent requirement was waived. We retrospectively reviewed our institutional HR-MRI database from January 2018 to June 2021. Patients who presented with CICAO on TOF-MRA or CTA and underwent both HR-MRI and DSA examinations were included in the study. The detailed inclusion criteria included (1) recanalization attempts were performed; (2) the time interval between HR-MRI and DSA was within 2 weeks; (3) HR-MRI cover the whole ICA from the bifurcation of the common carotid artery to the body of the corpus callosum; (4) the image quality of HR-MRI was adequate for evaluation; and (5) patients had one or more traditional atherosclerotic risk factors, such as hypertension, diabetes mellitus, hypercholesterolemia, and current cigarette smoking. The exclusion criteria included (1) pseudo-occlusion, defined as an extremely narrow residual lumen with a string-like or more normal distal lumen (14), or severe stenosis diagnosed by DSA; (2) history of surgery between HR-MRI imaging and DSA; (3) insufficient ICA coverage or uninterpretable images due to motion artifacts; and (4) nonatherosclerotic vasculopathies, such as vasculitis, dissection, or moyamoya disease.

Recanalization attempts were performed in symptomatic patients if the symptoms were attributed to the CICAO and hypoperfusion could be confirmed on MR perfusion-weighted imaging. For asymptomatic patients, considering that the generally accepted management of CICAO is conservative and the optimal therapeutic approach remains controversial, recanalization attempts were carefully decided after a multidisciplinary consensus meeting that included neurointerventionist, neurologist, and radiologist. Technical success was defined as thrombolysis in cerebral infarction (TICI) classification of 2b or 3 on postprocedural angiography.

### MR imaging protocols

All patients were examined using a 3.0T MR system (Siemens Skyra; Erlangen, Germany) with a 20-channel head/neck coil. The detailed protocols were as follows: (1) Compressed sensing TOF-MRA based on a research sequence and reconstruction prototype: repetition time (TR) 21 ms, echo time (TE) 3.49 ms, flip angle 18°, field-of-view (FOV) 220 × 200 mm^2^, matrix 368 × 334, slice thickness 0.4 mm, number of slabs 8. The acquired resolution was 0.6 × 0.6 × 0.6 mm^3^ and reconstructed to 0.4 × 0.4 × 0.4 mm^3^. The scan range extended from the bifurcation of the common carotid artery to the body of the corpus callosum. Data were reconstructed using 10 iterations of the modified fast iterative shrinkage-thresholding algorithm. (2) 3D T1-weighted SPACE (sampling perfection with application optimized contrast using different angle evolutions) sequence before and after contrast administration: TR/TE 900/4.2 ms, FOV 240 × 216 mm^2^ (covering from carotid artery bifurcation to all intracranial arteries), turbo-spin factor 43 echos, echo spacing 4.2 ms, and acquired resolution 0.75 × 0.75 × 0.75 mm^3^ (before November 2019). DANTE (delay alternating with nutation for tailored excitation) SPACE sequence was used after November 2019, and the acquired voxel size was 0.6 × 0.6 × 0.6 mm^3^. Contrast-enhanced 3D SPACE was started with an ~5-min delay after administration of 0.1 mmol/kg contrast agent (gadodiamide, GE Healthcare, Ireland). (3) Axial diffusion-weighted imaging (DWI): b-value 0 and 1,000 mm^2^/s, FOV 230 × 230 mm, section thickness 5 mm, and matrix 192 × 192. (4) Dynamic susceptibility contrast-perfusion weighted imaging (DSC-PWI) performed with a gradient-echo echo-planar sequence by the use of a bolus of 0.1 mmol/kg contrast agent (gadodiamide, GE Healthcare, Cork, Ireland): TR/TE 1,500/30 ms, FOV 230 × 230 mm, section thickness 5 mm, matrix 128 × 128, injection rate 4.5 ml/s, and 50 dynamic phases.

### Image reconstruction and analyses

Two neuroradiologists (with 6 and 4 years of experience), blinded to the DSA images, reviewed the HR-MRI images independently. Analysis of HR-MRI was mainly based on curved planar reformation. The observers were allowed to adjust the angle or projection to best display the characteristics of the occluded segment. For any disagreement, another senior neuroradiologist (with 10 years of experience) re-evaluated the images and assisted in reaching a consensus agreement.

**Figure 1:**
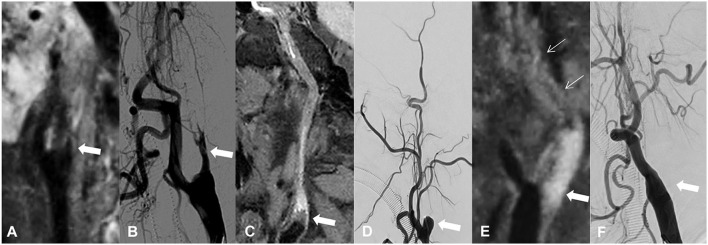
Classification of proximal stump in patients with CICAO. Arrows show the proximal stump condition on HR-MRI and DSA: **(A,B)** occlusion with tapered stump, **(C,D)** occlusion with blunt stump, and **(E,F)** occlusion with no stump.

The following variables were assessed on HR-MRI. (1) Occlusion with collapse, defined as the diameter of lumen distal to the occluded segments significantly decreasing to more than 50% of the contralateral normal lumen at the same level. (2) Number of occlusion segments, defined as the number of segments involved in chronic occlusion. ICA segments were evaluated according to the classification criteria proposed by Bouthillier et al. (15). (3) Occlusion length, categorized as ≤ 50 or > 50 mm. (4) The presence of high signal intensity (SI) in the occluded segment, defined as the SI in occluded lumen ≥150% SI of the normal-appearing vessel wall. (5) Occlusion of the cervical ICA, categorized as present or not. (6) Proximal stump condition, classified as a tapered stump, blunt stump, and no stump (4). Examples are shown in Figure 1. (7) Tortuosity of C1 segment, defined as at least one bend of >45° assessed throughout the occluded C1 segment. (8) Occlusion involving cavernous segment (C4), defined as occlusion localized in the C4 segment or long occlusion extended to the C4 segment. (9) Occlusion involving ophthalmic artery segment (C6) or above, defined occlusion localized in C6 and above, or long occlusion extended to the C6 segment or above.

### Statistical analysis

Quantitative data conforming to the normal distribution are represented as the means ± standard deviations, otherwise as the medians [interquartile range (IQR) is presented as the 25th and 75th percentile]. Categorical data were recorded as counts and percentages. The interreader reproducibility for assessing HR-MRI characteristics was evaluated using kappa analysis. To identify CICAO features that are associated with the technical success of recanalization therapy, the chi-square test or Fisher's exact test was used to compare groups of categorical data as appropriate. Variables reaching significance on univariable analysis (*P* < 0.05) were entered into the multivariable logistic regression analysis using a stepwise backward method after adjusting for age and gender. Odds ratios (ORs) with a 95% confidence interval (CI) were calculated. Then, a score-based prediction model was constructed using a regression coefficient-based scoring method (13). All statistical analyses were performed using the SPSS software version 22.0 (IBM Corp., Armonk, NY, USA). A two-sided *P*-value of 0.05 was considered statistically significant.

## Results

### Demographic information

**Figure 2:**
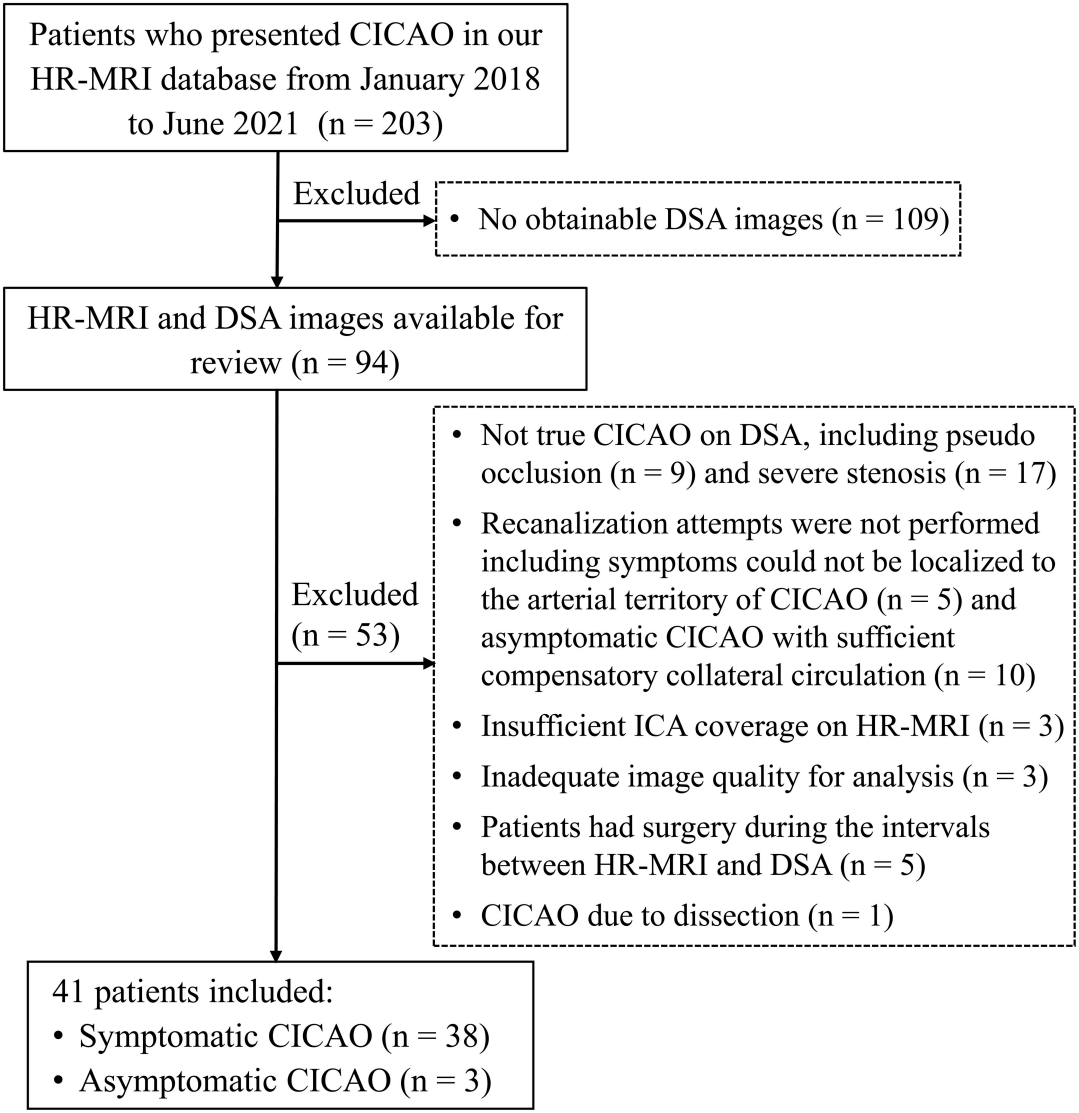
Flowchart of patient selection.

In total, 41 patients with CICAO (32 men, mean age 59.34 ± 9.69 years) who underwent endovascular recanalization attempts were included, including 38 symptomatic and three asymptomatic patients. Most symptomatic patients (85.4%) had a history of neurological events within 6 months. The three asymptomatic patients suffered from non-specific neurological symptoms like dizziness and headache, and hypoperfusion could be observed in all of them. The selection flowchart is detailed in Figure 2.

Successful recanalization was achieved in 26 (63.4%) patients. No significant differences were found between the success and failure groups regarding age, sex, risk factors, and time duration from the last neurological event. The detailed demographics, clinical features, and comparisons between the two groups are listed in Table 1. The 1-month procedural complication rate was 12.2% (5 of 41 patients), including acute ischemic events associated with distal embolization (*n* = 2), subarachnoid hemorrhage related to wire extravasation (*n* = 1), asymptomatic dissection (*n* = 1), and hyperperfusion syndrome with delayed nonfatal intracranial hemorrhage (*n* = 1). None of these complications resulted in severe disability or death. Asymptomatic reocclusion occurred in 7.7% (2/26) of patients with recanalization within 3–6 months after the procedure.

**Table 1:** Demographics and clinical characteristics for patients with or without technical success after recanalization attempts.

**Characteristics**	**Successful group (*n* = 26)**	**Failure group (*n* = 15)**	**Total (*n* = 41)**	***P* value**
Age (year) ^*^	61.23 ± 8.99	56.01 ± 10.30	59.34 ± 9.69	0.101
Male, *n* (%)	20 (76.9%)	12 (80.0%)	32 (78.0%)	1.000
Traditional risk factors				
Hypertension, *n* (%)	19 (73.1%)	11 (73.3%)	30 (73.2%)	1.000
Diabetes mellitus, *n* (%)	12 (46.2%)	6 (40.0%)	18 (43.9%)	0.702
Hyperlipidemia, *n* (%)	2 (7.7%)	2 (13.3%)	4 (9.8%)	0.615
Smoking, *n* (%)	9 (34.6%)	4 (26.7%)	13 (31.7%)	0.734
Coronary heart disease, *n* (%)	5 (19.2%)	1 (6.7%)	6 (14.6%)	0.388
Duration from last neurologic event				0.195
Any neurologic event ≤ 6 months, *n* (%)	24 (92.3%)	11 (73.3%)	35 (85.4%)	
Any neurologic event > 6 months, *n* (%)	1 (3.8%)	2 (13.3%)	3 (7.3%)	
No neurologic event, *n* (%)	1 (3.8%)	2 (13.3%)	3 (7.3%)	

* Data were expressed as mean ± standard deviation.

### Predictors on HR-MRI for technical success

The interreader agreement for evaluating HR-MRI features was moderate to excellent, with kappa values ranging from 0.74 to 0.94. There was also an excellent agreement between DSA and HR-MRI for the evaluation of stump condition, as well as the reversed flow of the ophthalmic artery (kappa: 0.85 and 0.89). The comparison of HR-MRI characteristics between patients with and without successful recanalization showed a significant difference in stump condition (*P* = 0.036), the number of occlusion segments (*P* = 0.013), and occlusion involving C6 segment or above (*P* < 0.001). In the patients' group that achieved technical success, the number of occlusion segments was smaller (median, 3.5 vs. 7), with a higher percentage of tapered stump morphology (70.0% vs. 28.6%) and fewer occlusions involving C6 segment or above (23.1% vs. 80.0%) compared with those who failed. No significant differences were found between the two groups in terms of occlusion with collapse, occlusion length, the presence of high SI, occlusion of proximal ICA, tortuosity of the C1 segment, and the occlusion involving the C4 segment. The comparison of HR-MRI characteristics between patients with or without technical success after recanalization attempts is summarized in Table 2.

**Table 2:** HR-MRI characteristics for patients with or without technical success after recanalization attempts.

**Characteristics**	**Successful group (*n* = 26)**	**Failure group (*n* = 15)**	**Total (*n* = 41)**	***P* value**
Left lesion, *n* (%)	15 (57.7%)	11 (73.3%)	26 (63.4%)	0.317
Occlusion with collapse, *n* (%)	7 (26.9%)	7 (46.7%)	14 (34.1%)	0.199
Number of occlusion segment^*^	3.5 (2,4)	7 (4,7)	4 (2.5, 6)	0.013
Occlusion length > 50 mm, *n* (%)	16 (61.5%)	12 (80.0%)	28 (68.3%)	0.305
Presence of high SI, *n* (%)	15 (57.7%)	7 (46.7%)	22 (53.7%)	0.495
Occlusion of cervical ICA, *n* (%)	20 (76.9%)	14 (93.3%)	34 (82.9%)	0.232
Stump condition, *n* (%)				0.036
Tapered	14 (70.0%)	4 (28.6%)	18 (52.9%)	
Blunt	3 (15.0%)	7 (50.0%)	10 (29.4%)	
No stump	3 (15.0%)	3 (21.4%)	6 (17.6%)	
Tortuosity of C1 segment >45°, *n* (%)	6 (23.1%)	3 (20.0%)	9 (22.0%)	1.000
Occlusion involving C4 segment, *n* (%)	19 (73.1%)	14 (93.3%)	33 (80.5%)	0.220
Occlusion involving C6 segment or above, *n* (%)	6 (23.1%)	12 (80.0%)	18 (43.9%)	<0.001

* Data were expressed as median (interquartile range presented as the 25th and 75th percentile). ICA, internal carotid artery; SI, signal intensity; C1 segment, cervical segment; C4 segment, cavernous segment; C6 segment, ophthalmic artery segment.

Multivariable logistic regression using the backward method showed that occlusion involving the C6 segment or above (OR = 0.036, 95% CI: 0.004 to 0.336, *P* = 0.004) and nontapered stump of proximal ICA including blunt and no stump morphology (OR = 0.064; 95% CI: 0.007 to 0.591, *P* = 0.015) were independent negative predictors for technical success in CICAO recanalization after adjusting for age and gender (Table 3).

**Table 3:** Predictors of technical success.

**Characteristics**	**Coefficient**	**Odds ratio (95% CI)**	***P* value**
Male	0.173	1.189 (0.084–16.795)	0.898
Age (year)	0.046	1.047 (0.923–1.188)	0.473
Number of occlusion segment ≥ 4	−0.659	0.517 (0.046–5.857)	0.595
Non-tapered stump of CICAO^*^	−2.755	0.064 (0.007–0.591)	0.015
Occlusion of C6 segment or above	−3.332	0.036 (0.004–0.336)	0.004

* Nontapered stump including blunt type and absence of stump morphology. ICA, internal carotid artery; CICAO, chronic internal carotid artery occlusion; C6 segment, ophthalmic artery segment.

### The clinical use of HR-MRI predictors for technical success

**Figure 3:**
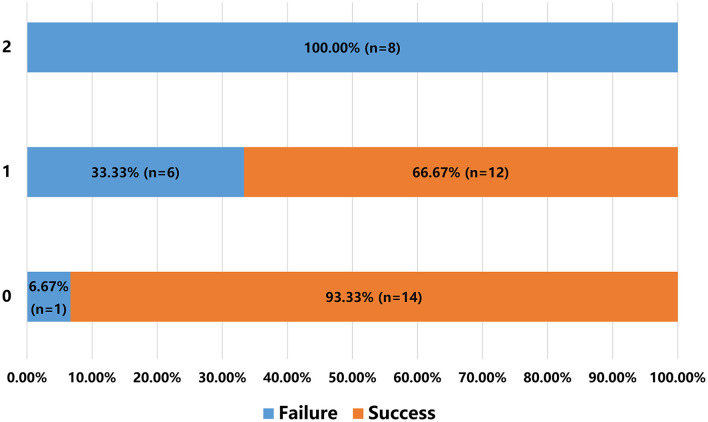
A scoring system to predict the success rate before recanalization in patients with CICAO. Y-axis shows each group of patients scoring 0, 1, or 2 points. X-axis shows percentage of patients who failed or succeeded in recanalization in each group.

**Figure 4:**
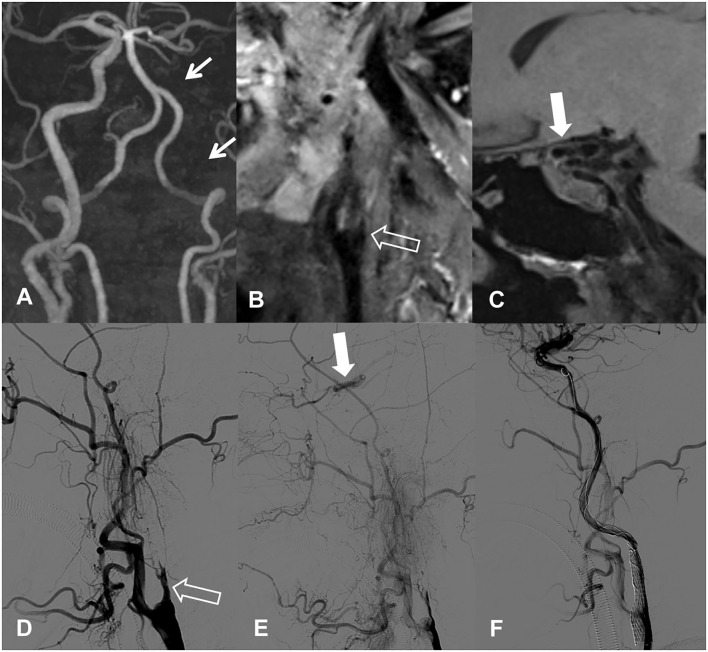
A 72-year-old female with right-sided limb weakness. **(A)** Long segmental occlusion of left internal carotid artery (ICA) is detected by TOF-MRA. **(B,C)** HR-MRI shows occlusion of proximal ICA with tapered stump and patent lumen of ophthalmic artery segment and above. This patient had scored as point 0 before recanalization. **(D,E)** Lateral images on DSA confirm the tapered stump condition and reversed flow from the ophthalmic artery. **(F)** Successful recanalization of the left ICA is achieved, followed by stent implantation.

**Figure 5:**
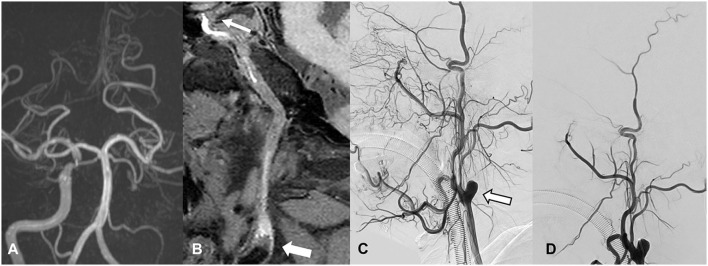
A 49-year-old man with progressive visual loss of left eye. **(A)** Long segmental occlusion of left internal carotid artery (ICA) is detected by TOF-MRA. **(B)** HR-MRI shows occlusion with blunt stump and involving ophthalmic artery segment and above. This patient had scored as point 2 before recanalization. **(C)** Lateral images of DSA confirm the blunt stump and no reversed flow of distal ICA. **(D)** The recanalization attempt is failed because catheter cannot pass the stump.

Using the independent predictors obtained above, a scoring system could be created to predict the success rate before CICAO recanalization. We assigned occlusion involving C6 segment or above and occlusion with nontapered stump one point, respectively, as a risk score according to their β coefficient in logistic regression analysis (Table 3). For patients who scored 0, 1, or 2 points, the success rates were 93.33% (14/15), 66.67% (12/18), and 0% (0/8), respectively (Figure 3). Representative cases are shown in Figures 4, 5 and Supplementary Figures S1 and S2.

## Discussion

Although considered as an effective treatment for CICAO (16), endovascular recanalization of CICAO still involves technical difficulties and potential complications. Sufficient preoperative assessment of visual clues for wiring across the occlusion is important to identify more suitable candidates for recanalization. In this study, we found that occlusions involving the C6 segment or above and nontapered stump were independent negative predictors for technical success. We further constructed a scoring system to estimate the success rate before endovascular recanalization for individual patients with CICAO.

It was generally agreed that the stump condition of occluded vessels, occlusion segments, and distal ICA reconstitution based on DSA were major determinants of technical success in patients with CICAO. Chen et al. reported a lower success rate in nontapered vs. tapered stumps (OR: 0.33; 95% CI: 0.13 to 0.82) (4). Hasan et al. showed that patients with longer occlusion length had a lower success rate, especially when it extended into the intracerebral ICA (17). Chen et al. and Mo et al. demonstrated that patent lumen distally with collateral filling from the ophthalmic artery, posterior communicating artery, and/or anterior cerebral artery was significantly important in improving the success rate (3, 4). However, DSA is an invasive and radiation-associated technique. Moreover, as a luminal imaging technique, a false-positive tandem occlusion might be suggested on DSA, or other etiologies of CICAO such as dissection might not be well differentiated, which therefore would significantly impact decision-making for some patients who should be excluded from endovascular recanalization.

As the most commonly used non-invasive techniques, CTA and TOF-MRA were reported useful for predicting the success rate of endovascular recanalization in CICAO (18). However, TOF-MRA and single-phase CTA may also overestimate the presence of an ICA occlusion due to false-positive findings (7, 8). In case of near-occlusion with or without full collapse and isolated intracranial or extracranial occlusion, CTA and MRA can “outrun” the arrival of a blood signal or contrast media and present as inadequate arterial angiography and the appearance of occlusion due to slow blood flow (8–10). This phenomenon limits the value of CTA and MRA in predicting technical success. Non-invasive imaging, which can better visualize the occlusion features, would be more helpful for screening potential candidates for recanalization therapy.

HR-MRI permits an excellent depiction of both the structural characteristics of the vessel wall and the vessel lumen and therefore is the first-line imaging tool to differentiate between different cervical arteriopathies. Recently, Zhang et al. showed that HR-MRI demonstrated superior accuracy to 3D-TOF-MRA for diagnosing CICAO and showed excellent agreement in the identification of occluded sites and proximal stump conditions compared with DSA (11). Moreover, HR-MRI provided additional information regarding the occluded segment, such as distal true lumen visibility, collapse, and vessel wall lesion components. Chai et al. demonstrated that HR-MRI could allow the identification of true ICA tandem occlusion in patients with an absence of a signal on MRA and identify suitable candidates for recanalization therapy (12). However, the predictive value of their method has not been validated due to the limited number of surgery cases (*n* = 13). In accordance with traditional DSA features like a tapered stump and reversed the flow of ophthalmic artery, we found that occlusions involving the C6 segment or above and nontapered stump on HR-MRI were independent negative predictors for technical success (4, 17, 19), which may be explained by the significantly increased difficulty of guide wire manipulation in patients with these factors. Theoretically, it is difficult to negotiate the wire through the long CICAO due to the variable vessel course, and it easily induces pseudoaneurysm formation and artery dissection. The success rate was lower in patients with a greater number of occlusion segments, as expected. However, the number of occlusion segments was not an independent predictor according to the multivariate analysis. We speculated that the impact of occlusion length on technical success might be mediated by the occlusion of the C6 segment or above.

No significant differences were found between the success and failure groups regarding occlusion with collapse and the presence of high SI in our study. We found it was difficult to describe the whole occluded segment with uniform high SI. Mixed high SI and isointensity in the occluded segment were more frequently encountered, indicating the existence of fresh and organized thrombus at the same time. Spontaneous recanalization and reformation of the thrombus in the occluded segment may contribute to the heterogeneous SI. A total of 14 (34.1%) patients with CICAO in our study had occlusion with collapse, which may suggest a long-time fibrosis occlusion or even congenital atresia of the vessel. It would be more difficult to find the true lumen and easier to form dissection when the guide wire passes through in these patients (20, 21). The frequency of occlusion with the collapse in the successful group showed a smaller trend than that of the failure group (26.9% vs. 46.7%), although no significant difference was found. Previously, Yan et al. quantitatively assessed the diameter of occluded ICA on HR-MRI and considered that the ipsilateral-to-contralateral diameter ratios of C1 (0.79) or C2 (0.70) could be used to predict the success rate of recanalization (13). In our study, the occlusion with collapse was defined as the diameter of lumen distal to occluded segments significantly decreased to ≥ 50% of the contralateral normal lumen at the same level, which was more stringent compared with theirs and may therefore lead to the relatively lower rate of occlusion with collapse, as well as inconsistent result. Further studies with quantitative analysis of vessel diameter and a large cohort of patients are still warranted.

We constructed a two-factor model based on HR-MRI findings to facilitate the identification of candidate patients. Patients who got 0, 1, and 2 points in our scoring system achieved 93.3%, 66.7%, and 0.0% successful recanalization, respectively. Some radiographic classification systems were reported previously but mainly based on DSA findings. Both Hasan et al. and Mo et al. divided the CICAO into four types (type A-D), consistent with 0 point (type A), 1 point (type B and C), and 2 points (type D) in our scoring system (3, 17). They reported the success rate as 100% and 90% in type A and 25% and 0% in type D, respectively. However, the rate of successful recanalization largely varied in types B and C (50–100%). A 100.0% success rate of type B (nontapered ICA stump and patent distal ICA) was reported by Hasan et al., but type B was rarely observed in our study (four of 41 patients, 9.8%), and successful recanalization was achieved in three (75.0%) of them. As a result, we consider that this scoring system might have greater clinical significance for screening patients with CICAO who were very suitable (point 0) or unsuitable (point 2) for recanalization procedure.

This study has a few limitations. First, this was a retrospective study with relatively small sample size. Endovascular recanalization in asymptomatic patients is still controversial. Therefore, these patients were very carefully selected in our center. The ongoing CREST-2 trial (carotid revascularization endarterectomy vs. stenting trial) would be very helpful for identifying the best strategy for stroke prevention in these patients. In the future, a prospective and large cohort of patients is still needed to validate current findings. Second, the protocols of 3D T1-SPACE were inconsistent in this study. We used the DANTE SPACE sequence after November 2019, as it can provide better signal suppression for the slow blood flow and cerebrospinal fluid, thus improving the wall contrast. Finally, a follow-up study is needed to assess the long-term efficacy after recanalization.

## Conclusion

HR-MRI may be a useful tool to identify candidates for endovascular recanalization in patients with CICAO. Occlusions involving C6 segment or above and nontapered stump were independent negative predictors for technical success.

## Data availability statement

The raw data supporting the conclusions of this article are available upon reasonable request to the corresponding authors.

## Ethics statement

The studies involving human participants were reviewed and approved by Ethics Committee of the First Affiliated Hospital of Nanjing Medical University. Written informed consent for participation was not required for this study in accordance with the national legislation and the institutional requirements.

## Author contributions

XZ: conceptualization, methodology, software, investigation, formal analysis, and writing—original draft. CZ: data curation and writing—original draft. YZC: visualization and investigation. CQS: investigation. HBS: resources and supervision. SSL: conceptualization, funding acquisition, supervision, writing—review and editing, and resources. SL: conceptualization, supervision, writing—review and editing, and resources. All authors contributed to the article and approved the submitted version.

## Funding

This study was supported by the National Natural Science Foundation of China (Grant Number: 82171907 to SSL).

## Conflict of interest

The authors declare that the research was conducted in the absence of any commercial or financial relationships that could be construed as a potential conflict of interest.

## Publisher's note

All claims expressed in this article are solely those of the authors and do not necessarily represent those of their affiliated organizations, or those of the publisher, the editors and the reviewers. Any product that may be evaluated in this article, or claim that may be made by its manufacturer, is not guaranteed or endorsed by the publisher.
