# Supplementary material for: High-resolution magnetic resonance imaging for predicting successful recanalization in patients with chronic internal carotid artery occlusion
Source: Front Neurol. 2022 Sep 1;13:1003800. doi: 10.3389/fneur.2022.1003800 (PMC9475072; doi:10.3389/fneur.2022.1003800)
Supplement: Supplementary file 1 [file Table_1.DOCX]

**Supplementary Figure legends**


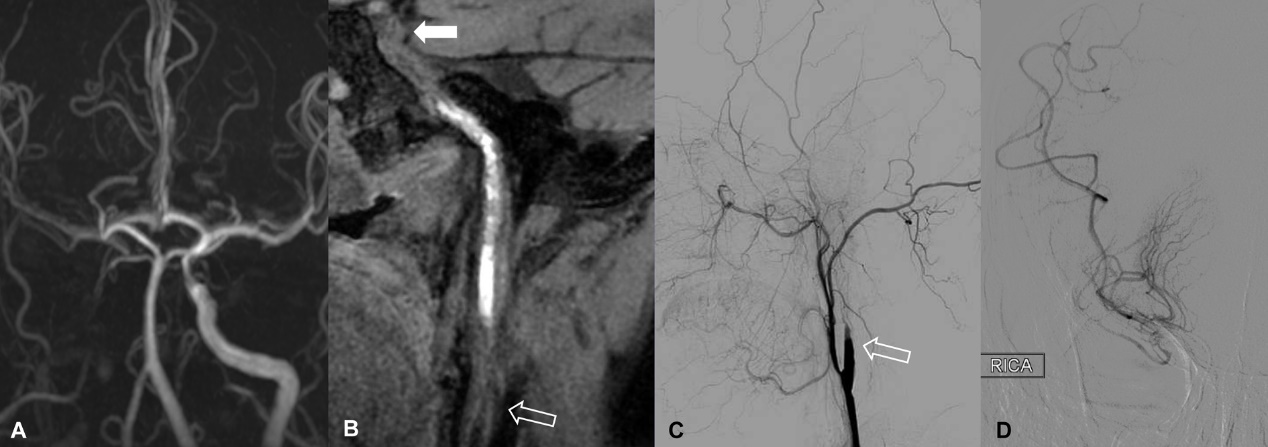


**Figure S1.** A 43-year-old man with left-sided limb weakness. (A) Long segmental occlusion of right internal carotid artery (ICA) is detected by TOF-MRA; (B) HR-MRI shows occlusion with tapered stump and involving ophthalmic artery segment and above; This patient is scored as point 1 before recanalization; (C) Lateral images of DSA confirm the tapered stump and no reversed flow of distal ICA; (D) The guide wire and micro catheter could enter the right middle cerebral artery through the occluded segment. But the recanalization attempt was failed and terminated considering the risk of thrombus falling off.


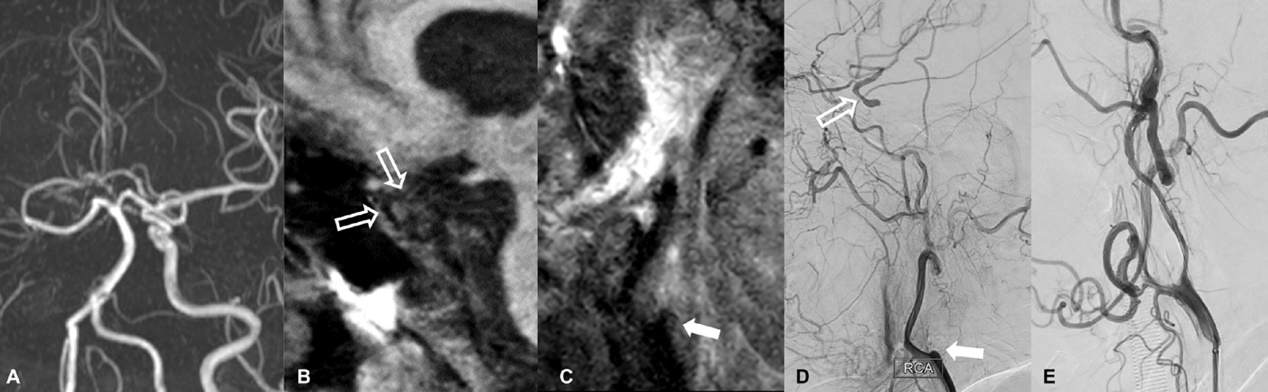


**Figure S2.** A 68-year-old man with left-sided upper limb weakness. (A) Long segmental occlusion of right internal carotid artery (ICA) is detected by TOF-MRA; (B) HR-MRI shows occlusion with blunt stump and patent lumen of ophthalmic artery segment and above; This patient is scored as point 1 before recanalization; (C) Lateral images of DSA confirm the blunt stump and reversed flow from ophthalmic artery; (D) Successful recanalization of right ICA is achieved followed by stent implantation.
